# Testing Activity Monitors’ Effect on Health: Study Protocol for a Randomized Controlled Trial Among Older Primary Care Patients

**DOI:** 10.2196/resprot.5454

**Published:** 2016-04-29

**Authors:** Zakkoyya H Lewis, Kenneth J Ottenbacher, Steve R Fisher, Kristofer Jennings, Arleen F Brown, Maria C Swartz, Elizabeth J Lyons

**Affiliations:** ^1^ University of Texas Medical Branch Division of Rehabilitation Sciences Galveston, TX United States; ^2^ University of Texas Medical Branch Department of Preventive Medicine and Community Health Galveston, TX United States; ^3^ University of Texas Medical Branch Department of Physical Therapy Galveston, TX United States; ^4^ University of California Los Angeles Department of Medicine Division of General Internal Medicine/Health Services Research Los Angeles, CA United States; ^5^ University of Texas Medical Branch Department of Nutrition and Metabolism Galveston, TX United States

**Keywords:** physical activity, cardiovascular disease, prevention, activity monitors, technology, primary care, 5 A counseling, intervention

## Abstract

**Background:**

Cardiovascular disease is the leading cause of mortality in the United States. Maintaining healthy levels of physical activity is critical to cardiovascular health, but many older adults are inactive. There is a growing body of evidence linking low motivation and inactivity. Standard behavioral counseling techniques used within the primary care setting strive to increase motivation, but often do not emphasize the key component of self-control. The addition of electronic activity monitors (EAMs) to counseling protocols may provide more effective behavior change and increase overall motivation for exercise through interactive self-monitoring, feedback, and social support from other users.

**Objective:**

The objective of the study is to conduct a three month intervention trial that will test the feasibility of adding an EAM system to brief counseling within a primary care setting. Participants (n=40) will be randomized to receive evidence-based brief counseling plus either an EAM or a pedometer.

**Methods:**

Throughout the intervention, we will test its feasibility and acceptability, the change in primary outcomes (cardiovascular risk and physical activity), and the change in secondary outcomes (adherence, weight and body composition, health status, motivation, physical function, psychological feelings, and self-regulation). Upon completion of the intervention, we will also conduct focus groups with the participants and with primary care stakeholders.

**Results:**

The study started recruitment in October 2015 and is scheduled to be completed by October 2016.

**Conclusions:**

This project will lay the groundwork and establish the infrastructure for intervention refinement and ultimately translation within the primary care setting in order to prevent cardiovascular disease on a population level.

**Trial Registration:**

ClinicalTrails.gov NCT02554435; https://clinicaltrials.gov/ct2/show/NCT02554435 (Archived by WebCite at http://www.webcitation/6fUlW5tdT)

## Introduction

### Cardiovascular Disease and Physical Activity

In the United States, more than 85 million adults have at least one type of cardiovascular disease (CVD) [1]. It is the most deadly disease in the United States (accounting for 1 in 3 deaths) and the world [1,2]. By 2020, the American Heart Association (AHA) strives to reduce this number by 20% [3]. It is well established that lifestyle behaviors, such as physical activity (PA), are related to CVD [4-9]. Therefore, intervening on PA is an important step toward meeting this goal [10]. The Centers for Disease Control recommendation for primary and secondary prevention of CVD is 30 minutes of PA a day, 6-7 days per week [7]. However, analysis of the Behavioral Risk Factor Surveillance System reports the prevalence of older adults meeting this recommendation only reaches 39.3% [11]. Lack of motivation to exercise appears to be a barrier that impedes meeting the recommendation for many older adults [12].

A promising strategy for increasing motivation for exercise is promotion by primary care providers [13-16]. The United States Preventive Services Task Force (USPSTF) developed the 5 A’s counseling intervention to be used in the primary care setting [17]. The components of 5 A’s stand for assess, advise, agree, assist, and arrange. Five A’s is a comprehensive and validated counseling technique to elicit behavioral change during a typical medical visit [10,17]. This counseling is based on the Self-Determination Theory, which targets perceptions of autonomy, competence, and relatedness to increase autonomous motivation, that is, motivation that is volitional and internal rather than external [18,19].

Most primary care physicians use at least one 5 A’s measure with their patients [20]; however, comprehensive behavioral counseling or complete 5 A’s counseling is not routinely conducted [15,20]. Even if proper 5 A’s counseling was done routinely, providing knowledge and professional support alone is insufficient for changing behavior [21]. Emphasizing self-control through self-monitoring of behavior is an effective behavioral change technique (BCT) when combined with other techniques, such as shaping knowledge and social support [21,22]. Use of technology is a promising method for delivering these techniques [16]. The AHA recommends both 5 A’s counseling and technology-based interventions to improve cardiovascular health for individuals at moderate risk for disease [10].

PA interventions that utilize self-monitoring technologies have been shown to be efficacious [23-25]. Moreover, self-monitoring technologies are feasible to implement with 5 A’s counseling [23] and, thus, may help prevent the onset and progression/or progression of chronic diseases [26]. A self-monitoring technology commonly used is a pedometer [23,27-32]. Pedometers are cost-effective activity monitors that provide a simple method of promoting self-control via self-monitoring of steps [33]. Pedometer use is associated with improvements in PA, weight, and blood pressure [27-29]. These improvements may be sustained to prevent chronic disease risk factors (measured by the Framingham risk calculator) [31]. In a previous primary care intervention, individuals wearing a pedometer had significant increases in PA compared to usual care [34]. Ideally, technologies for preventing CVD should provide self-monitoring, education, personalized feedback, and should also be customizable [35]. Since standard pedometers cannot provide all of these features, they may not be optimal for CVD prevention.

More advanced pedometers, or electronic activity monitors (EAMs), are readily available on the market (estimated 3.3 million units sold in 2014) [36] and offer many features that standard pedometers do not. Like pedometers, EAMs count total steps, but they can also measure duration and intensity of PA. EAMs can sync wirelessly to an application (app) on a smart device (phone or tablet). The data it collects are displayed clearly and concisely through the app. EAMs are programmable in that the individual can set his or her personal activity goals [37]. Lyons et al [38] found that EAMs include numerous behavioral techniques commonly associated with PA change including: self-monitoring, feedback, goal-setting, planning, social support, social comparisons, commitment, instructions on how to perform a behavior, and information on consequences [39,40]. EAMs have the potential to change behavior alone, but they will likely be more successful if combined with structured engagement [41], such as a research intervention. Providing an EAM in addition to 5 A’s counseling shows promise in preventing CVD because: (1) an EAM can deliver counseling components that may not have been targeted by the physician [42]; (2) it provides adaptive PA goals based on progress, which is more effective than a static exercise prescription [43]; and (3) it can improve perceptions of relatedness by providing social support from other users.

### Specific Aims

**Figure 1:**
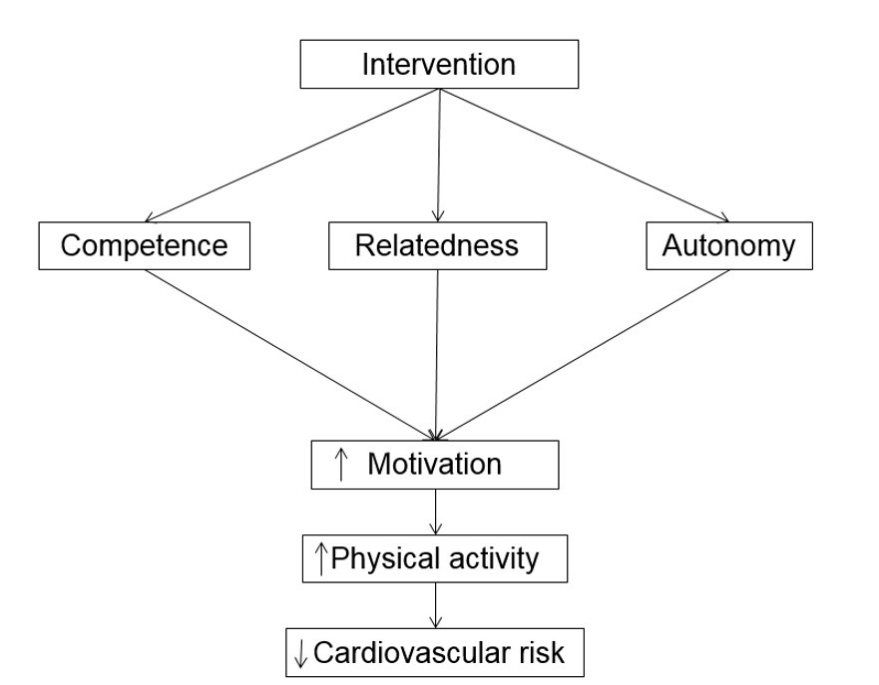
Conceptual framework of the intervention.

Testing Activity Monitors’ Effect (TAME) on health (NCT02554435) will implement and evaluate a 12 week intervention that incorporates 5 A’s counseling and self-monitoring with an activity monitor among older primary care patients. Participants (n=40) will be randomized to receive 5 A’s counseling and an EAM system or 5 A’s counseling and a standard pedometer. Figure 1 shows the conceptual framework of TAME health (also outlined in Table 1). As a follow-up to the intervention, separate focus groups with research participants and primary care stakeholders will be conducted to further gauge the intervention’s feasibility and acceptability.

**Table 1:** Conceptual constructs underpinning the research design.

Theoretical construct		BCT^a^	Intervention components		
			5 A’s counseling (both groups)	Pedometer	EAM
**Self-Determination Theory**					
	Autonomy	Goal-setting/intention formation	X		X
		Provide information on consequences of behavior in general	X		X
		Barrier identification/problem solving			X
		Action planning	X		X
	Competence	Provide feedback on performance	X	X	X
		Provide instruction	X		X
		Teach to use prompts/cues			X
		Self-control: Prompt self-monitoring of behavior		X	X
	Relatedness	Facilitate social comparison			X
		Social support	X		X

^a^ BCTs described are associated with Self-Determination Theory constructs based on previous research [22,44-46].

## Methods

### Preliminary Studies

Several preliminary studies have shaped the proposed intervention. In a preexperimental pilot study, we recruited 10 sedentary older adults (55-79 years of age) into a six week intervention in which they received an EAM (UP by Jawbone), a mini tablet, and weekly counseling. Adding an EAM to the PA intervention was both feasible and acceptable within this older adult population that already used smart devices. There were nine out of the 10 participants that agreed or strongly agreed with the following statements: “I would continue to wear the monitor”; “I felt very confident using the tablet”; “The UP app encouraged me to view my steps”; and “It was convenient for me to use the UP app”. As a follow-up, we are completing a pilot intervention to determine changes in PA outcomes within this same population using an UP24 by Jawbone. We will compare PA outcomes between those who received the intervention to wait-list controls. Among the 38 participants to complete the intervention at this time, we found that nearly all used social support features of some kind. Several participants (8/38) even commented over 50 times across the 12 week period.

In addition to conducting PA interventions, we analyzed the content of EAMs. We evaluated 13 different EAMs and coded them for present BCTs [38] and validated their measurements of steps over the span of a week compared to research-grade activity monitors. We found that EAMs commonly incorporate important BCTs [38] and they reasonably estimate the number of steps per day.

The research team, led by ZHL, also conducted a systematic review of PA interventions that utilized EAMs [37]. This review evaluated different intervention methodologies and EAMs used by researchers. Findings suggested that EAM interventions produced equivocal results when compared to behavioral counseling alone. However, the devices investigated did not provide social support and used apps with fewer behavior change techniques than provided in Jawbone’s app. Compliance outcomes indicated that devices were regularly worn and utilized by participants [37]. Overall, our work to date suggests that EAMs are motivational devices that are acceptable for use by older adults and can change PA behavior in conjunction with behavioral counseling.

### Protocol

This protocol has been approved by the University of Texas Medical Branch (UTMB) Institutional Review Board (Protocol 15-0014, Version 5, 12/02/15) and is registered on a website (NCT02554435). This protocol also follows SPIRIT reporting guidelines (see Multimedia Appendix 1). The TAME health study design is displayed in Table 2.

We intend to recruit older primary care patients (n=40) to participate in the 12 week TAME health study. Primary eligibility criteria include: age (55-74 years), physically inactive (less than 60 min/week of PA), body mass index between 25-35, in good health as measured by the PA Readiness Questionnaire Plus (Par-Q+) [47], and access to a smart device. Eligibility criteria were determined based on our previous work using EAMs in this population. A smart device is operationally defined as a mobile phone or tablet using the iOS operating system or Android 4.3 or later. Reports suggest that 45% of adults over the age of 65 own a smart device [48]. Individuals will be excluded from the study if participation in PA is inadvisable by their doctor, they are involved in another PA intervention currently or within the past 6 months, utilized an EAM in the past 6 months, are unwilling to travel for scheduled visits, currently taking medications that affect body composition, a current smoker, report of alcohol or drug problem, institutionalizations for psychiatric illness within the last year, or do not consent. As advised in the Par-Q+ [47], prospective participants with comorbid conditions are eligible if they provide a letter from their physician stating that it is safe for them to participate in PA.

**Table 2:** TAME health time frame.

		Study period				
		Enrollment	Allocation	Post allocation		Close-out
Study week		-12	0	1	12	12
**Enrollment**:						
	Eligibility screen	X	X			
	Informed consent	X				
	Allocation		X			
I**nterventions**:						
	5 A’s counseling		X	X		
	Pedometer			X	X	
	EAM			X	X	
**Assessments**:						
	Age (date of birth)	X	X			
	Gender	X	X			
	Education		X			X
	Cardiovascular risk		X			X
	PA	X	X	X		X
	Weight	X	X			X
	Body mass index	X	X			X
	Body composition		X			X
	Blood pressure		X			X
	Exercise motivation		X			X
	Health status and quality of life		X			X
	Physical function		X			X
	Psychological feelings		X			X
	Resting pulse		X			X

### Recruitment and Screening Procedures

Participants will be recruited from two clinics affiliated with a large university-based health care system. Recruitment started in October 2015 and will continue until all participants are enrolled. Prospective participants will be recruited from flier postings in waiting rooms and in patient rooms, direct solicitation from a recruitment table in the lobby, and through recruitment letters mailed to their home.

Patient eligibility will be determined by using a screening script. Screenings will be completed in person at the recruitment table or over the phone. Once patients are deemed eligible, the researchers will obtain informed consent. This will be done promptly after screening for eligibility at the recruitment table for patients screened in person. For patients screened over the phone, a researcher will arrange a meeting with the patient at their primary care clinic to obtain informed consent.

After obtaining informed consent, an assessment visit will be scheduled. Reminders about the orientation meeting will be sent via email monthly (for those recruited prior to the data collection start date of January 1, 2016) and one week prior to the scheduled meeting. In addition, participants will receive a phone call reminder the day before the meeting. Enrollment into the intervention is postponed until after January 1, 2016 to minimize seasonal variations in PA [49].

### Intervention Procedures

#### 5 A’s Counseling

All participants will receive brief PA counseling following the USPSTF 5 A’s model [17,18]. The counseling provided in this intervention is referred to the “5A-S model” for it emphasizes the importance of self-control. The counseling components included: assess, advise, agree, assist, arrange, and self-monitor. The brief PA counseling will occur during the private assessment visit at the participant’s primary care clinic, before randomization. An experienced PA counselor with a background in exercise physiology and training in motivational interviewing will conduct the counseling. To facilitate transition into clinical practice, participants will go through counseling at baseline and will be instructed to self-monitor with their assigned device for the subsequent 12 weeks. After counseling, the researcher will provide the participant with an exercise prescription to follow. A phone call will be arranged with the participant a week after the counseling to assess progress and resolve technical issues. Table 3 provides a description and an example of how each 5 A’s component will be operationalized in the current study.

**Table 3:** Counseling components.

	Description^a^	Example
Assess	Ask about/assess behavioral risk	Review steps per day and minutes of PA per day from research-grade monitor
Advise	Advise participant to increase their PA to meet healthy levels	Compare current PA level to CDC guideline Define moderate and vigorous PA Suggest new PA goal
Agree	Reach agreement with the participant about appropriate weekly PA goal and a long term goal	Set weekly step goal Active planning of step goal Set long term PA goal
Assist	Teach behavioral change strategies	Identify social support Identify barriers Provide PA prescription
Arrange	Arrange a follow-up appointment to assess progress and any issues that arise	Schedule phone call in 1 week Review health changes Go through the “Agree” step on new weekly goal

^a^ The descriptions are modified from Whitlock et al [17].

#### Grouping the Participants

All participants will be given an activity monitor to encourage self-regulation of PA. After counseling, participants will be randomized to one of the two groups: pedometer or EAM group. A random number generator available on the Internet will be used to randomly allocate group assignment [50]. Assignment into the EAM group will be selected randomly by the generator using the following settings: 1 set, 5 unique numbers, numbers range from 1 to 10, and sorted lowest to highest. This process will be repeated for 3 more sets of numbers ranging from 11-20, 21-30, and 31-40. Each number represents a participant identification number selected to be in the EAM group. Group assignment will be written on a piece of paper wrapped in foil and carbon paper and concealed inside an envelope prior to group allocation. A blinded researcher will prepare all of the envelopes. After group allocation, participants will be given all intervention materials and detailed instructions on all intervention components. For participants in the EAM group, the researcher will also test the monitor and the participant’s smart device to ensure they are working properly. Due to the nature of the intervention, the participants and the accessor will not be blinded to group assignment after randomization.

#### Pedometer Group

Participants will be provided with a digital pedometer (Digi-walker CW-700/701, YAMAX, San Antonio, TX). The pedometer records total number of steps (steps), activity time (hour/minute), distance walked (mile), and calories burned (kcal). The device stores this information every day and up to the previous 7 days. Weekly totals for steps, activity time, distance walked, and calories burned are stored for the current and previous week. The pedometer also displays a clock and an informative graph that estimates the number of calories burned based on the number of steps [51]. Participants will be instructed to log their daily steps, activity time, and distance walked measured by the pedometer in an activity diary.

#### Electronic Activity Monitors Group

All participants will be given an EAM (UP24 by Jawbone, San Francisco, CA) and the corresponding UP app on their smart device. UP24 by Jawbone is widely used and is one of the top selling EAMs [52]. Furthermore, UP24 implements the most BCTs compared to other available EAMs and provides vital techniques that are missing in the counseling. In total, Jawbone UP24 provided 27 BCTs in 2014, but with continual software updates, this number is likely higher [38]. Some examples include goal setting on behavior and on a health outcome, self-monitoring of behavior, emotional social support, providing instructions, providing information on consequences, social comparison, prompts, and focus on past success [38]. For these reasons, UP24 was selected for the intervention.

The UP app provides information related to PA, diet, weight, sleep, and socialization. Participants will not be counseled on improving sleep or diet, but they are additive features of the app. The home screen of the app provides immediate feedback to the user on their activity for the day. On the home screen, the wearer also has access to their activity feed, which displays all their activities and their teammates’ activities. All activities in the app are stored forever so the user can review all past activity. In addition, the app allows the user to set goals, record activity, and review activity trends. The user can also set reminders for activity and learn more information about their health from the Smart Coach tailoring messaging feature [53]. The specific features throughout the app are described in Multimedia Appendix 2 (see Multimedia Appendix 2).

All participants will be given a deidentified account with an anonymous name and icon to use in the app. We have chosen to use board game pieces names and icon pictures (eg, Battleship, Cat, Hotel) based upon suggestions from previous participants and enjoyment of this system in our pilot studies. Participants will be required to add other participants as their “teammate” in order to utilize the socializing features within the app. Only PA information will be required to be visible by their teammates. Participants will be able to customize their account if they wish to share any other behaviors with their teammates. Participants that do not consent to have their deidentified information shared with other participants will be ineligible and dropped from the intervention. In order to view the participant’s activity, the participants will also add the research account as a teammate. The researchers will not socialize or interact with the participants through the app; it will be for surveillance purposes only. Participants will be given a trouble-shooting guide to help with any issues with the app. The researcher will also review and resolve any technical issues reported during the “Arrange” phone call.

### Assessment Procedures

The primary purpose of this study is to investigate the feasibility and acceptability of a low-intensity intervention that combines 5 A’s counseling and an EAM system. We will also compare the effects of these high-tech activity monitors to low-tech ones (pedometers) on PA and other cardiovascular risk indicators (Framingham risk calculator, fitness). The study includes two assessments conducted at baseline and 12 weeks. Upon initial recruitment at the clinic, participants will provide informed consent and schedule their first assessment visit. Approximately 7 days before their initial assessment, participants will be given a baseline questionnaire and a research-grade activity monitor (discussed below) to wear for a 7 day baseline period. Participants will return to their primary care facility to complete the assessment. At 11 weeks, participants will be given a follow-up questionnaire and the research-grade monitor to wear for 7 days. At 12 weeks, participants will return to their clinic to complete assessments identical to baseline. As reimbursement for taking part in this study, all participants will receive a US $25 gift card at the end of the 12 week assessment, and at that time participants also forfeit their EAM or pedometer device.

The primary outcomes of interest are feasibility and acceptability. Feasibility will be operationalized in several ways. Number of days logged for activity, interactions with other users in the mobile app, and additional use of the app will be abstracted using procedures we have previously pilot tested successfully. Acceptability will be measured using self-report (modeled on items previously developed) [54], responses, and focus groups conducted after completion of the intervention. The primary physiological variables of interest include cardiovascular risk indicators and PA. Secondary outcome variables are anthropometrics, body composition, blood pressure, exercise motivation, health status and quality of life, physical function, psychological feelings, resting pulse, and self-regulation. All physiological study variables are listed in Table 4.

**Table 4:** Study variables.

Variable		Measure	Subscales	Alpha statistics
**Primary variables**				
	CVD risk	Framingham nonlaboratory risk score calculator	Age: yrs; body mass index: *kg*/m^2^; systolic blood pressure: mmHg	
		Six minute walk test	Distance walked in 6 minutes: ft	
	PA	Sensewear armband (BodyMedia, Pittsburgh, PA)	Minutes of METs ≥ 3 (7 days of measurement); steps per day	
**Secondary variables**				
	Anthropometrics	Stadiometer (Seca Corp, Hamburg, Germany); scale (Tanita, Arlington Heights, IL)	Height (baseline only): cm; weight: kg; body mass index: *kg*/m^2^	
	Body composition	Tape measure (Singer, China)	Waist circumference: cm; hip circumference: cm; waist to hip ratio	
	Blood pressure	Sphygmomanometer (Omron BP742N, Lake Forest, IL)	Systolic blood pressure: mmHg; diastolic blood pressure: mmHg	
	Demographics	Self-report	Age: yrs; sex; race/ethnicity; education	
	Exercise motivation	Behavioral Regulation in Exercise Questionnaire-2	Intrinsic, identified, introjected, extrinsic, amotivation	.73-.86
	Health status and quality of life	36-Item Short Form Health Survey	Physical functioning, social functioning, physical role limitations, emotional role limitations, mental health, energy/vitality, pain	.76-.90
	Physical function	Short physical performance battery	Repeated chair stands, balance, semitandem stand, side-by-side stand, tandem stand, 8 feet walk	
		PROMIS SF v1.2-Physical function 8b	Upper extremities, lower extremities, central regions	
	Psychological feelings	Psychological Need Satisfaction in Exercise Scale	Perceived competence, perceived autonomy, perceived relatedness	>.90
	Resting pulse	Sphygmomanometer (Omron BP791T, Lake Forest, IL)	Heart rate: bpm	
	Self-regulation	Rovinak et al scale [55]	Exercise goals, exercise plans	.87-.89

### Cardiovascular Risk

CVD risk is determined by measuring 10 year risk of cardiac event from the Framingham nonlaboratory equation [56] and from fitness measured by a six minute walk test [57]. The six minute walk test is an additive assessment of CVD risk because cardiorespiratory fitness is the result of chronic PA and is more strongly related to clinical outcomes [58]. The Framingham equation has been used to estimate CVD risk in previous studies [31,59]. It requires a combination of self-report and objective measures [56,60]. Self-report measures include sex, age, treatment of hypertension, smoking status, and diagnosis of diabetes. Objective measures included blood pressure and body mass index [56,60].

The six minute walk test requires participants to walk a 100 feet course continuously for 6 minutes. It has been validated in older adults to measure physical endurance (0.71<r<0.82) [57]. Percentile norms on the test have been established for active older adults [61].

### Physical Activity

PA will be measured with a SenseWear Armband. The armband is worn on the upper arm for 7 days. SenseWear quantifies PA by measuring expended kcals/week, minutes of activity a day, and steps per day. There is a mean error < 5% in measuring total minutes of PA with the SenseWear armband [62]. To properly program the armband, age, gender, and body mass index (BMI) will be collected at enrollment. These variables will be formally measured at a study allocation, described below.

### Secondary Outcomes

We will also assess anthropometrics, body composition, blood pressure, resting pulse, demographic information, health status and quality of life, physical function, exercise motivation, self-regulation, and psychological feelings toward exercise.

Anthropometrics include height (cm), weight (kg), and BMI (*kg*/m^2^). To ensure accurate measurement of anthropometrics, height and weight will be measured to the nearest 0.1 unit. Participants will be instructed to remove their shoes and any bulky clothing before measurement. Height will be measured using a portable stadiometer [63] and weight will be measured using a calibrated electronic scale [64]. The same equipment will be used at both clinics. The electronic scale will be transported in a cushioned carrying case to ensure it remains calibrated. BMI will be calculated by converting height into meters and squaring that value, then dividing weight by this value [65].

Body composition will be estimated based on the waist circumference (cm), hip circumference (cm), and waist-to-hip ratio. The waist will be measured at the smallest circumference of the torso, while the hip will be measured at the largest circumference at or below the anterior superior iliac spine [66]. The waist-to-hip ratio will be calculated by dividing the waist circumference by the hip circumference [66]. The average from three measurements of anthropometrics and body composition will be used.

Blood pressure and resting pulse will be measured by a sphygmomanometer (Omron BP742N, Lake Forest, IL) [67]. Blood pressure will be measured after the participant is seated for at least five minutes and before any other measurements are taken. The sphygmomanometer is validated for clinical and personal use according to the European Society of Hypertension International Protocol [67].

Demographic information will be collected at both assessments. This information includes date of birth (age), race/ethnicity, educational level, comorbid conditions, and medication use. The 36-Item Short Form Health Survey will be used to estimate health status and quality of life. Subscales of this instrument include physical functioning, social functioning, physical role limitations, emotional role limitations, mental health, energy/vitality, and pain [68].

Physical function will be measured objectively and subjectively. Physical function will be objectively assessed using the short physical performance battery. Total functional capacity is based on a composite score from the following subtests: repeated chair stands, balance (semitandem stand, side-by-side stand, tandem stand), and 8 feet up walk [69]. Physical function will be subjectively assessed using the PROMIS Short Form v1.2-Physical function 8b [70].

Several self-report measures will be used to estimate exercise motivation, self-regulation of exercise, and psychological feelings toward exercise [55,71,72]. Each measure includes subscales. Exercise motivation consists of intrinsic motivation, identified, introjected, external regulation, and amotivation [71]. Self-regulation consists of exercise goals and exercise plans [55]. Psychological feelings toward exercise consist of perceived competence, perceived autonomy, and perceived relatedness [72].

### Focus Groups

We will conduct several focus groups with research participants at the end of the intervention to further evaluate feasibility and acceptability. These sessions will consist of 5-8 individuals and last for 1.5-2 hours. A trained professional will facilitate all of the focus groups. Groups will be separated based on the intervention modality and two groups will be completed for each study arm. The focus groups will explore the perceived effectiveness of the 5 A’s counseling and the monitoring device. Participants will also be asked questions pertaining to their overall feelings toward the intervention and their thoughts of the intervention being implemented through their primary care physician.

Focus groups following the same structure, but with primary care stakeholders, will also be conducted at the conclusion of the intervention. Stakeholders (n=20) will consist of medical doctors, nurses, medical residents, physician assistants, and other staff personnel. Stakeholders will be recruited through coordination with the Department of Internal Medicine and Family Medicine at the clinics used in the study. All stakeholders will consent to be a part of the focus group and will be given the opportunity to wear the UP24 for one month. During the focus groups, stakeholders will be asked questions related to their perception of the intervention. Materials and equipment from the intervention will be provided and preliminary results of the intervention will be presented. The goal of the focus groups is to elicit reactions regarding the feasibility and acceptability of including this intervention into their primary care clinic. Stakeholders will also be asked about their familiarity with 5 A’s counseling and the extent of its use in practice. Stakeholders will complete a brief questionnaire before and after the focus group. In addition, stakeholders that wear the UP24 for one month will complete a feasibility questionnaire upon returning the monitor.

### Statistical Analyses

The Statistical Package for the Social Sciences (version 20) will be used to perform all quantitative statistical analyses. Analyses will be completed in two ways: first using the intent-to-treat principle (primary analysis) and then only with adherers to the intervention (secondary analysis for the purpose of informing further intervention refinement). The alpha-level to determine significance is set at .05. The specific statistical procedures, by research aim, are described below.

### Aim 1

Feasibility findings will be primarily descriptive and used as a metric for improvement when compared to similar studies. For example, days worn will be compared to other EAM studies, whereas attrition will be compared to other primary care-based studies. Rather than determine dichotomous feasibility or not, we will use relative feasibility to determine areas of focus for intervention refinement; which is addressed in the follow-up questionnaire and in focus groups. Comparisons of adherence between EAM and pedometer groups is covered under Aim 2 below and the procedures for analyzing focus groups will be identical to procedures outlined in Aim 3 below.

We hypothesize that the intervention will be feasible among several improvement metrics including ≥ 70% days the monitor is worn [37], < 20% attrition [73], and < 8 moderate or higher adverse events (< 20% of participants reporting an adverse event) [74-76]. Hypotheses based on other feasibility metrics include a low frequency of technical difficulties and a high frequency of app usage. Additionally, the intervention will be accepted among participants based on self-report questionnaires and focus group responses. We also hypothesize that the EAM group will show higher feasibility and acceptability compared to the pedometer group.

### Aim 2

Descriptive analyses will be conducted using means and frequencies for baseline characteristics. These characteristics include age, gender, race/ethnicity, educational level, CVD risk, PA, fitness, weight, BMI, blood pressure, pulse, wait and hip circumference, physical function, exercise motivation, health status, psychological feelings, and self-regulation. Mean group differences will be examined using independent samples *t* tests for normally distributed data and nonparametric tests (Kruskal-Wallis and Mann-Whitney U) for nonnormally distributed data. Differences in variable frequencies will be examined using chi-square tests. Little’s Missing Completely at Random test will be performed to determine whether outcome data are missing at random.

Analysis of covariance will be used to test the postintervention difference at 12 weeks (PA, CVD risk, fitness) between the two groups. The covariates in the analysis will include baseline values of the dependent variable, clinic location, and any variables significantly different between groups at baseline. A blinded statistician will conduct analysis on the primary outcome variables (PA, CVD risk, fitness). This same procedure will be used to assess differences in weight, BMI, body composition, health status, motivation, physical function, psychological feelings, and self-regulation. Group mean values for adherence variables at 12 weeks (retention rate, days the monitor worn) will be analyzed by an independent *t* test.

The primary purpose of this study is to evaluate its feasibility and acceptability; therefore, the analyses described in this section are exploratory and no prespecified power calculation is needed. Although this is a pilot study and it is not powered to detect significant difference in small-to-moderate effects, we hypothesize that the EAM group will demonstrate greater improvement in all of the aforementioned variables compared to the pedometer group.

### Aim 3

NVivo 11 Pro (QSR International) will be used to perform qualitative analyses. Thematic analysis will be conducted to analyze the focus groups [77]. Codes will be developed prior to the focus groups. Additional coding will be processed while the focus groups are conducted, adding codes based on new data. After including all study participants, the completed code list will be used to code transcripts of all groups. The PRECIS tool will also be used to estimate the intervention’s potential success in real clinical app [78].

### Power Calculation

Although the aim of the intervention is to improve behavioral and health outcomes related to cardiovascular risk, the primary purpose of this study is to investigate the feasibility of intervention components and study procedures and to inform a larger intervention. This is necessary in research related to CVD [79]. For these reasons, a sample size of 40 was deemed appropriate to test the intervention. The sample size of 40 is able to detect an increase of 1000 steps from baseline to follow-up, which is below the minimum expected increase in steps from pedometer-based interventions [80]. The findings from this pilot study will be used to establish initial effect sizes and inform the power and sample size estimates for future larger trials.

### Limitations

The 5 A’s counseling is meant to be delivered by a primary care physician [17]. A recent review of A-counseling found that patients want all counseling components conducted by their physician [81]. Our study is limited in that the PA counselor will provide all of the counseling. This study is also limited to more affluent individuals who can afford a smart device. However, if this study proves feasible, it will inform subsequent larger studies that will provide smart devices and counseling conducted by physicians. This study is also limited to the areas of greater Galveston and Harris Counties, TX. Although this study will not be nationally generalizable, we anticipate that it will include a diverse population.

Several potential limitations will be explicitly measured and addressed by the pilot study design. For example, the older adult participants may dislike aspects of the app or EAM, or compliance may be limited by misunderstandings. Furthermore, it may be insufficiently powered to detect all significant differences in study outcomes and it will not evaluate maintenance of PA.

### Data Management

Participants will be thoroughly informed of study procedures and give informed consent prior to any data collection. All data and participant information will be kept in a locked file cabinet or stored as a deidentified file on a secured computer network. Data and participant information will be transported from the clinics to the office in a passcode protected folder or on an encrypted flash drive. Data will be linked with an identification number and will be stored separately from all personal information. Only authorized members of the research team will have access to data. Due to the small sample size, a data monitoring committee will not be utilized. Instead, the Principal Investigator will conduct data safety monitoring weekly.

Participants in the EAM group will be given deidentifiable accounts to be used by the UP app. The accounts will not provide names, images, or personal information of the participants. UP app accounts made by stakeholders, as part of their involvement in the focus groups, will not be deidentified and will include any personal information the individual discloses in the app (ie, name, images). Accounts will only be viewable by other study participants and the research team.

## Results

TAME health is currently in the recruitment phase. Recruitment started in October and will continue until all participants are enrolled. As of December 3, 2015, a total of 8 participants have been enrolled. The intervention will start after January 1, 2016. The intervention component of TAME health is expected to be done by the end of June 2016. At that time, focus groups will be conducted and the results will be analyzed. The entire study is scheduled to be completed by October 2016. Any protocol modifications and study results will be updated on a website (NCT02554435).

## Discussion

There is a need for scalable, relatively inexpensive, and low-intensity interventions that could be delivered across a large population [82]. After the completion of the study and focus groups, we will refine the intervention based upon findings as well as qualitative and quantitative participant feedback. This project will lay the groundwork and establish the infrastructure for a successful program of related research. Ultimately, we hope to use the results of our research program to implement health care programs that utilize technology to provide effective behavioral interventions on a public health scale, improving population CVD morbidity and mortality [10,16].

## Appendix

Multimedia Appendix 1 SPIRIT Guideline Checklist.

Multimedia Appendix 2 Feature within the UP app by Jawbone.

Multimedia Appendix 3 Grant proposal reviewer comments.

## Abbreviations

AHA: American Heart Association
app: application
BCT: behavioral change technique
BMI: body mass index
CVD: cardiovascular disease
EAM: electronic activity monitor
PA: physical activity
Par-Q+: Physical Activity Readiness Questionnaire Plus
TAME health: Testing Activity Monitors’ Effect on health
USPSTF: United States Preventive Services Task Force
UTMB: University of Texas Medical Branch
