# Supplementary material for: Testing Activity Monitors’ Effect on Health: Study Protocol for a Randomized Controlled Trial Among Older Primary Care Patients
Source: JMIR Res Protoc. 2016 Apr 29;5(2):e59. doi: 10.2196/resprot.5454 (PMC4867768; doi:10.2196/resprot.5454)
Supplement: Multimedia Appendix 2 [file resprot_v5i2e59_app2.pdf]

All screen shot photographs were taken on September 30th, 2015. The UP app is updated regularly and some aspects may be different than those available at the time of reading.

UP APP HOME SCREEN

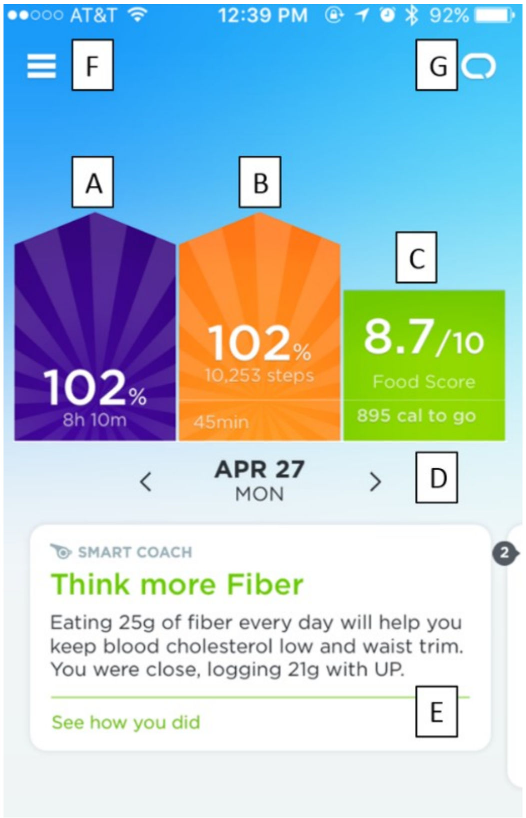

A. Sleep activity from the previous night. From the home screen the user can view their total sleep time and the percent of their sleep goal they met.

B. Physical activity progress for the day. From the home screen the user can view how many steps they've taken, how many minutes they were active, and what percent of their step goal is met.

C. Dietary activity for the day. From the home screen users can see the score of their food source and how many more calories they can consume.

D. Displays the date for the activity shown. The user has the option to go forward or backward to view other days.

E. The UP Smart Coach provides information and tips to improve the user's health. The user can swipe to the right to view more advice from the Smart Coach.

F. This takes the user to the main menu where they can review all their activity, reset goals, view activity trends, view their team, create duels between other teammates, review messages from teammates, view partnering apps with UP, and view the UP Marketplace where users can purchase other health devices that partner with UP.

G. This takes the user to their device information where they can view their battery life, start sleep or activity.

UP GOAL SETTING, RECORDING, AND TRACKING

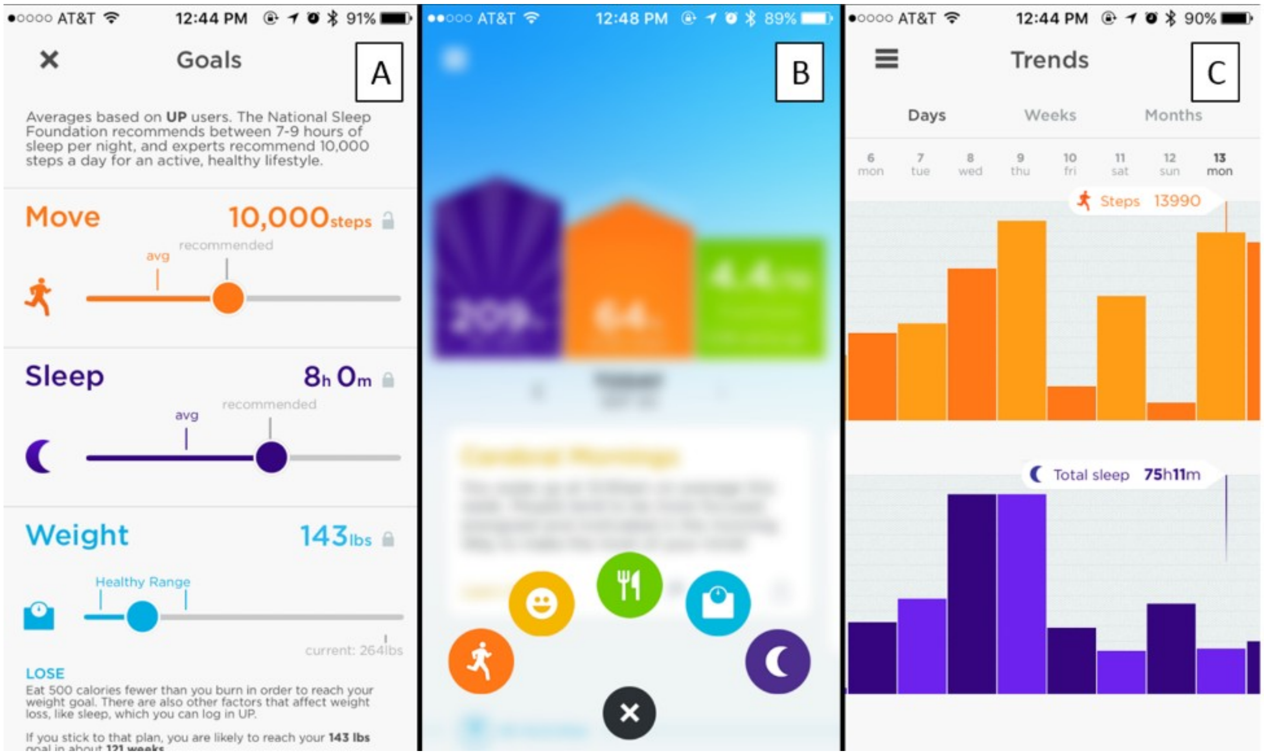

Image

A. Users can set their own daily goals for steps and sleep as well as long-term weight goal. The app provides recommendations based on public health agencies and comparisons based on the average activity from UP users. The app also suggests a healthy range for weight based on the user's BMI.

B. Users can manually report their activity. From left to right the user can report their physical activity, feelings, food intake, weight, and sleep.

C. The user can view their sleep, physical activity, diet and weight trends by day, week, or month.

PHYSICAL ACTIVITY

The UP24 monitor measures PA objectively and sends the information to the UP app via Bluetooth. The band measures the number of steps per day, distance walked, calories burned,

active time, percent of goal achieved, and sedentary time. The app displays a graph which depicts the level of activity throughout the day. Any activity that was manually entered or recorded in stop watch mode will appear on the graph as icons (Image 3A). When the user reviews their PA for the day in the app they also receive feedback on their progress toward their step goal for the past 7 days (Image 3B).

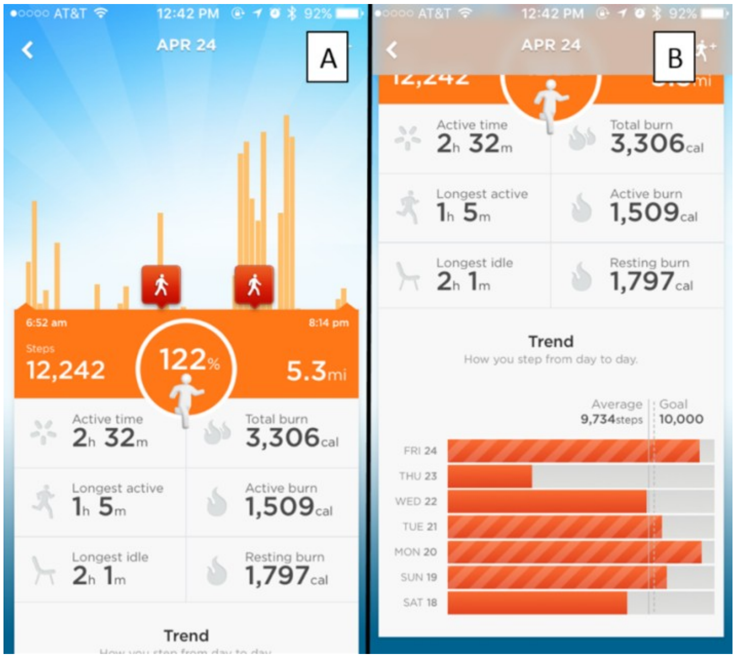

Image

Stopwatch mode is a feature that allows the user to specify an exercise activity. The user can start and stop stopwatch mode by pressing the button on the monitor or within the app. Once the user completes an exercise in stopwatch mode they can specify within the app the exercise they completed. The user must select the specific type of activity from a list of activities (e.g. walking, biking, Zumba, etc.) or select "other" if the exercise was not listed. Then the user must select their effort level. The specific effort levels include: easy, moderate, in the zone, difficult, and GUT BUSTER! The effort level is also depicted graphically to help select the appropriate level. If the user forgot to start the stopwatch mode before they started the exercise they can record in

the app later (Image 4A). They will need to provide the same information described above, the start time, and the duration of the exercise. In addition, the app can detect PA patterns that appear to be exercise. It will appear in their activity feed and will ask the user if they want to log the activity (Image 4B).

Idle alerts are a unique feature of the UP24 system (Image 4C). The user can program the monitor to vibrate after a pre-specified amount of idle time. The specified times range from 15 minutes to 2 hours with 15 minute increments. The monitor will only vibrate if the wearer was idle for the specified time, if they are not idle for an extended period of time the alert will not go off. When setting up the idle alerts, the user must also specify when the alerts will be active to ensure that alerts will not go off while the user is sleeping. Idle alerts can be enabled and disabled at the user's discretion.

In addition to the idle alerts, the user can program activity alerts (Image 4D). The app will send notifications about the wearer's step progress after a specified number of steps. The progress updates can be scheduled to appear after every 1,000 and up to 15,000 steps. The user can also program when to receive a move summary, which reports the total number of steps per day. Activity alerts can be enabled and disabled at the user's discretion.

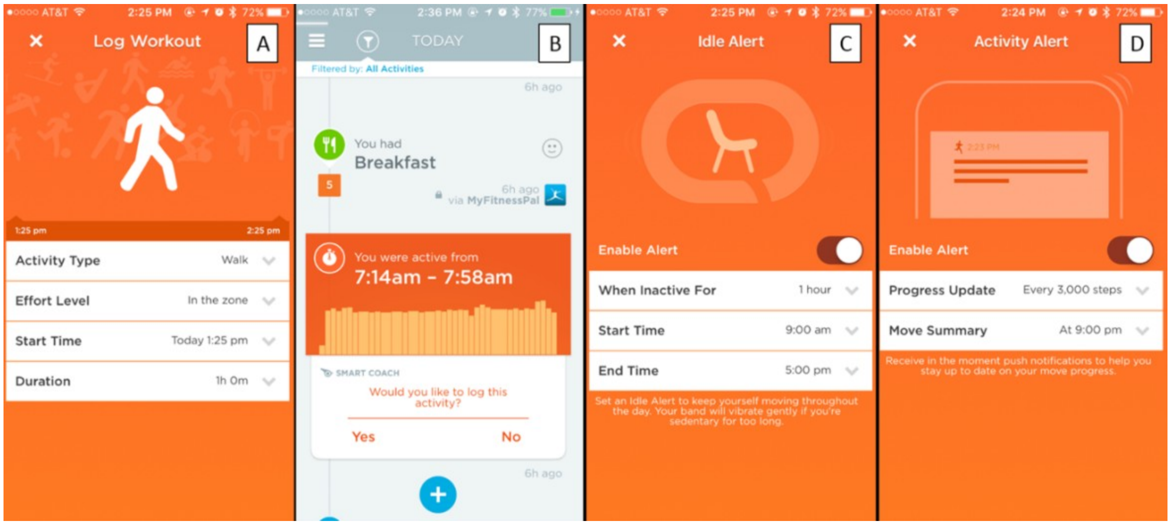

A
Log Workout
Activity Type
Walk
Effort Level
In the zone
Start Time
Today 1:25 pm
Duration
1h 0m
B
TODAY
You had
Breakfast
via MyFitnessPal
You were active from
7:14am - 7:58am
SMART COACH
Would you like to log this activity?
Yes
No
C
Idle Alert
Enable Alert
When Inactive For
1 hour
Start Time
9:00 am
End Time
5:00 pm
Set an Idle Alert to keep yourself moving throughout the day. Your band will vibrate gently if you're sedentary for too long.
D
Activity Alert
Enable Alert
Progress Update
Every 3,000 steps
Move Summary
At 9:00 pm
Receive in the moment push notifications to help you stay up to date on your move progress.

Image

SLEEP

Similar to PA, sleep is measured objectively by the monitor. The user puts the monitor into sleep mode by pressing a button on the monitor or directly in the app. This process is similar to setting activity mode. The next morning, the app displays the total sleep time, percent goal achieved, sound sleep time, light sleep time, the number of times the user woke up, as well as the amount of time it took to fall asleep, how many minutes the user was awake in bed, and how long the user was in bed. The app also displays a graph that illustrates the amount of time awake, in sound sleep, and in light sleep during the night (Image 5A). Also, like PA, the app provides a chart of the 7-day trend (Image 5B). If the user forgets to log their sleep, the app may detect a possible sleep pattern. It will appear in their feed and will ask the user if they want to log their sleep.

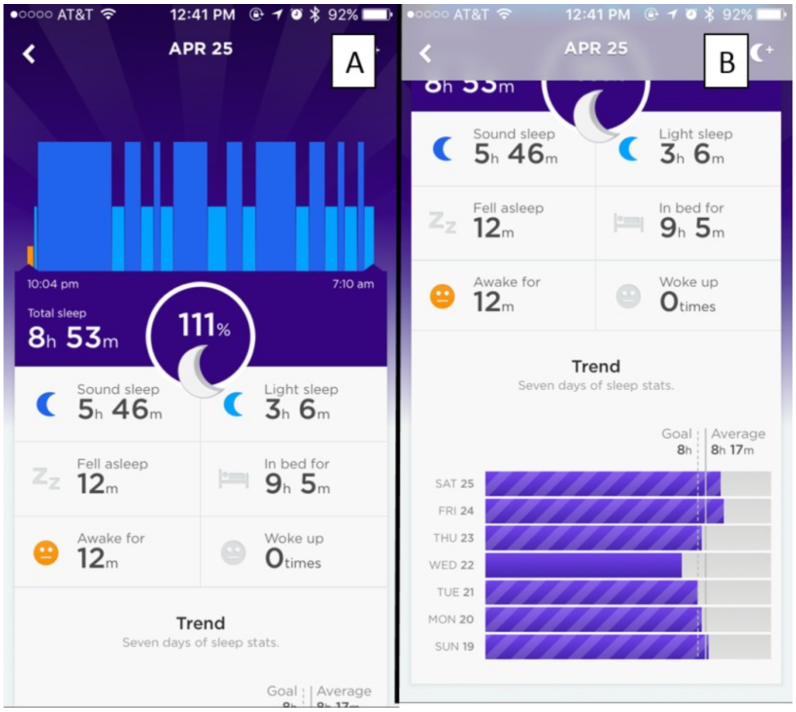

Image

The user can also program the monitor into “power nap” mode if they take a nap during the day. This process is similar to setting it into sleep mode at night. An additional sleep feature is the smart alarm. The user can program the monitor to vibrate when it is time to wake up. Within the app, the user must set the alarm time and a “smart alarm window” up to 30 minutes before the alarm time. The monitor vibrates during the window time and at the alarm time. The user can set the alarm to repeat on any desired day and the user can program multiple smart alarms.

DIETARY INTAKE

The app allows users to log their dietary intake directly into the app or in one of their partnering apps. The user can log food by searching for a specific food item, scanning the barcode of a food item, or searching through nearby restaurant menus (Image 6A). The user can modify the quantity of food to match how much food they consumed. As the user enters food the app displays how many calories they consumed earlier that day, how many calories are in the meal they are logging, and how many calories they have left to consumed based on the expected burned calories. The user has the option to take a picture of the food as part of the diary. Once the food is entered the app will display a score for their diet, the amount of calories consumed compared to the estimated calories burned, and the amount of nutrients consumed (Image 6B). The Food Score ranges from 0 to 10 with higher numbers representing healthier foods based on the nutrient value. The nutrients are broken down into “all you can eat” (fiber, unsaturated fat), “don’t overdo it” (other carbohydrates, protein), and “pass on these” (sugar, saturated fat, sodium, and cholesterol) (Image 6C). The app will also display a word map of the most common

foods consumed in the past 7 days.

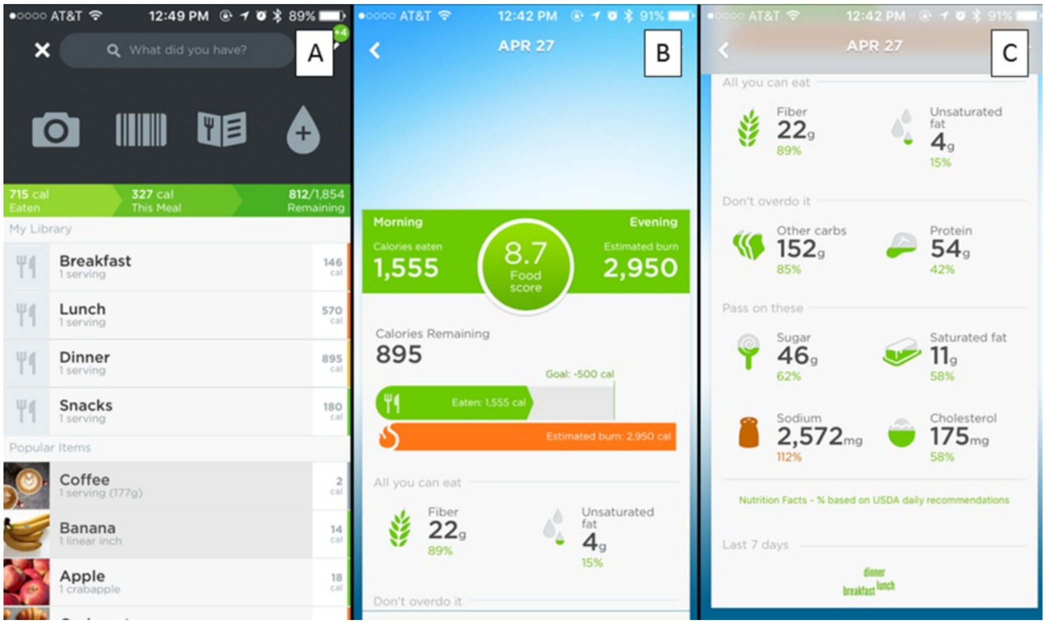

WEIGHT

Users can enter their weight into to app to track their weight change. If the user uses a compatible wireless electronic scale their weight will automatically update in the app. The app displays the weight graphically to illustrate the weight change (Image 7).

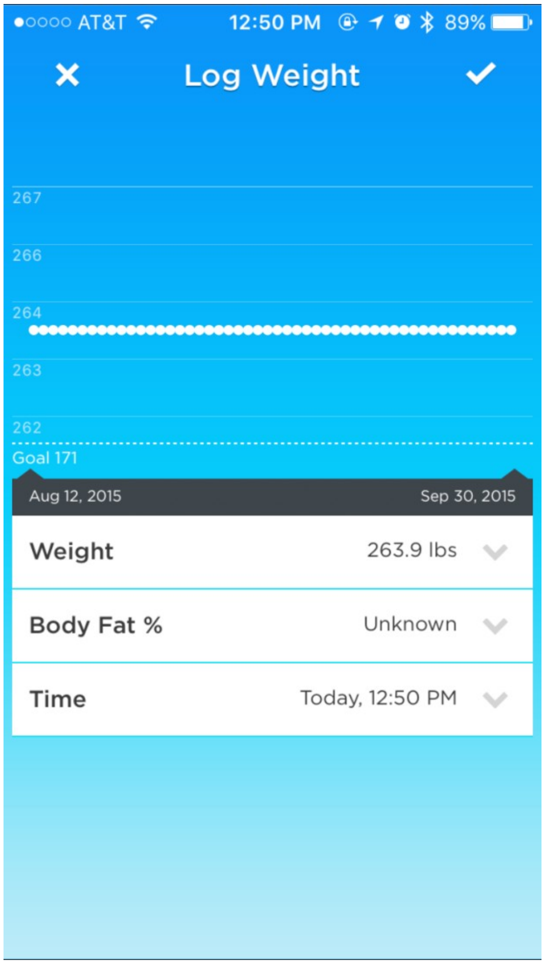

Image

SMART COACH

The Smart Coach provides the user with information and tips daily on all aspects of health. The information is both general (Image 8A) and individualized (Image 8C). The user can choose to "learn more" and the Smart Coach will send them to a link with more reading material (Image 8B, 11D).

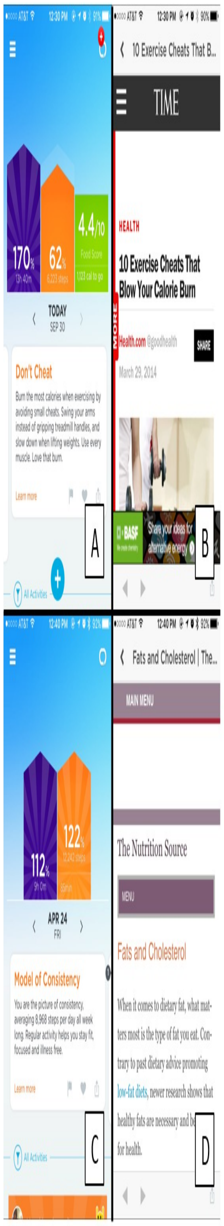

Image

The Smart Coach is also a motivational tool that tracks the user’s progress and encourages them to meet their goal. Smart Coach will offer a “Today I Will” based on the user’s activity. The “Today I Will” is a commitment the user makes with Smart Coach to accomplish a goal for that

day related to PA, sleep, or diet. The “Today I Will” appears at random but will repeat daily, once accepted, until the user doesn’t accept the challenge (Image 9).

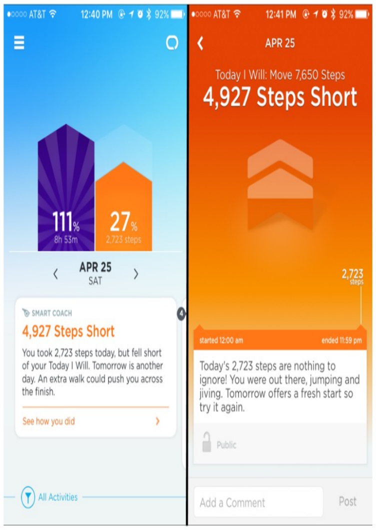

Image

The Smart Coach also appears throughout the user’s activity feed to provide encouragement and advice. Some specific examples of Smart Coach feedback is displayed in Image 10.

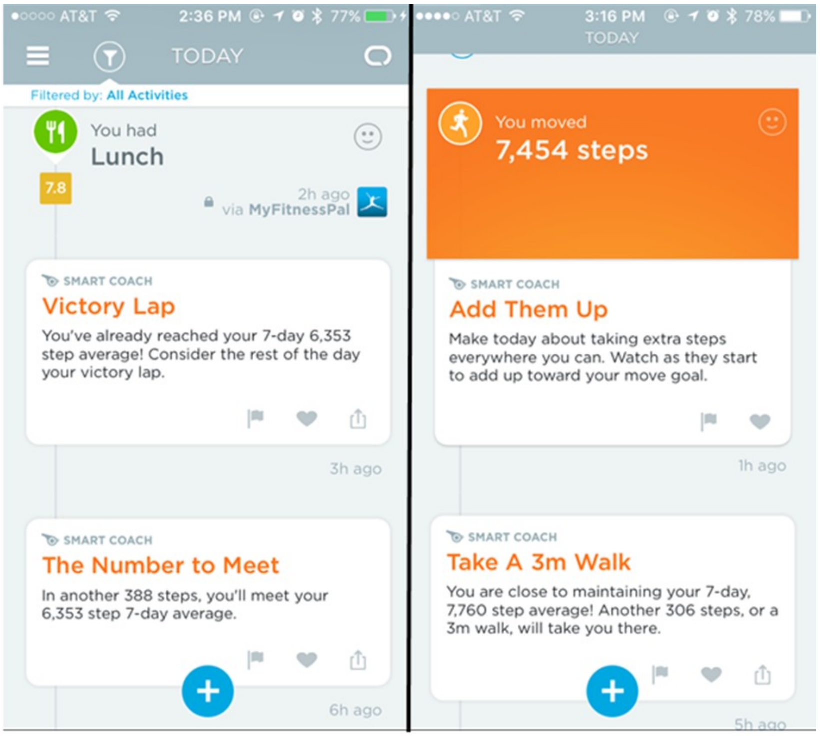

Image

REMINDERS

The user can program personalized reminders with the app (Image 11). The band will vibrate and the wearer will receive a notification alert from the app when the reminder is scheduled to go off. The reminders can be scheduled for a bedtime, workout, meal, or pill. There is also a “custom” option for the user to customize their reminder. The user can program the reminder to repeat on desired day and time. The user can also personalize the reminder by giving it a name.

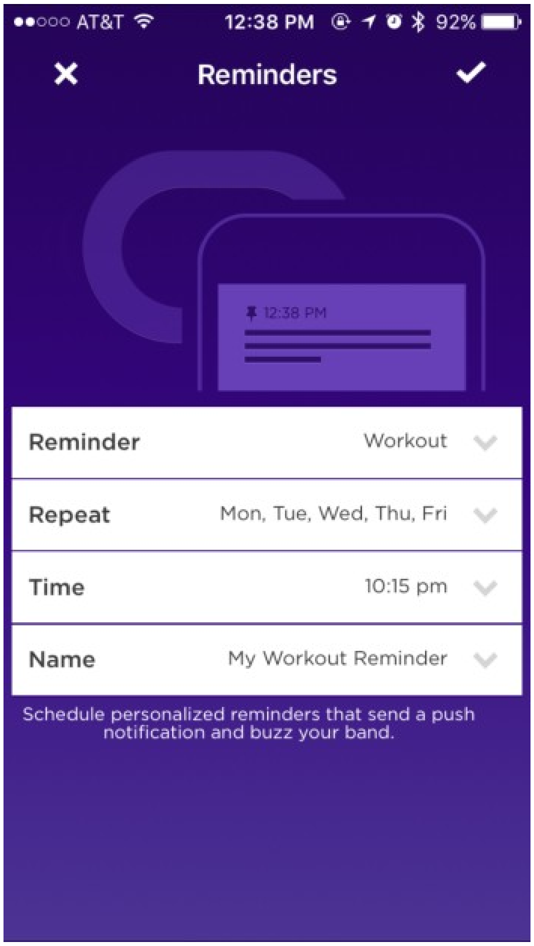

Image

SOCIALIZATION

The app includes several features that allow for socializing between UP users. The user can invite their friends to the app to join their team. The user can view their teammate's activity in their feed or the user can go to their UP page. Users can comment or "like" their teammate's activity (Image 12A) to provide social support. Users can view how they compare to their teammates through the leaderboard. The leaderboard displays the total steps from the past 7 days and ranks teammates from highest to lowest number of steps. Users can directly compete with one another through duels (Image 12B). Duels may last for 24 hours, 3 days, or 1 week. The user creating the duel can decide to make the public to all teammates of both duelers or private

between the two duelers. One user creates the duel and invites another teammate to join. The teammate can accept or decline the invitation. The user with the most steps after the designated time is the winner.

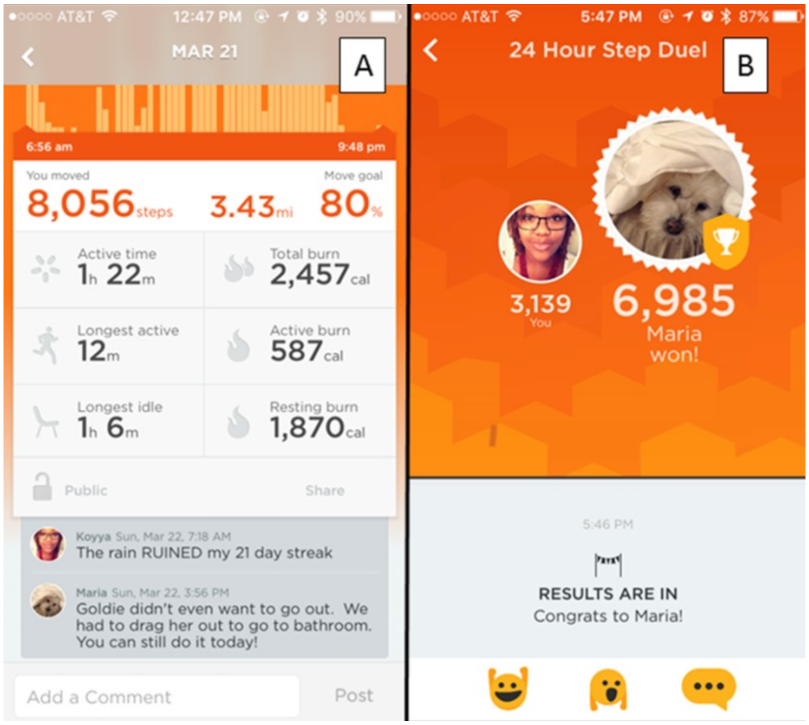

Image

The app also allows for interactions with the app through "feelings". Users can express how they are feeling for the day through an emoticon (Image 13). The designated "feelings" include: totally done, exhausted, dragging, meh, good, energized, pumped UP, or amazing! The user can also customize their feeling.

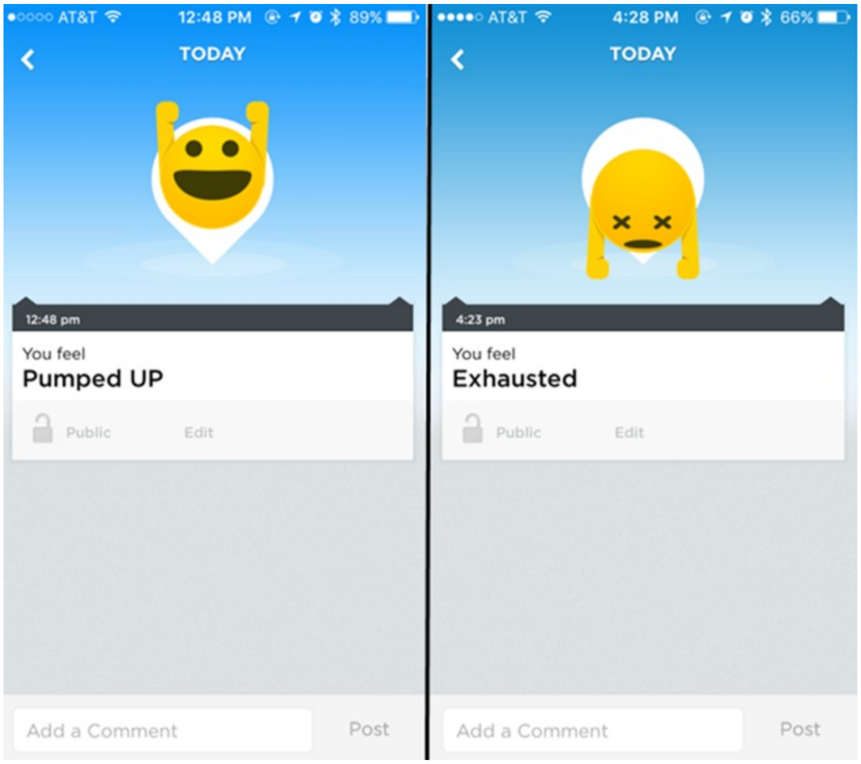
AT&T 12:48 PM 89%
TODAY
12:48 pm
You feel
Pumped UP
Public
Edit
Add a Comment
Post
AT&T 4:28 PM 66%
TODAY
4:23 pm
You feel
Exhausted
Public
Edit
Add a Comment
Post

Image
