# Supplementary material for: Testing Activity Monitors’ Effect on Health: Study Protocol for a Randomized Controlled Trial Among Older Primary Care Patients
Source: JMIR Res Protoc. 2016 Apr 29;5(2):e59. doi: 10.2196/resprot.5454 (PMC4867768; doi:10.2196/resprot.5454)
Supplement: Multimedia Appendix 3 [file resprot_v5i2e59_app3.pdf]

Reviewer Comments on Grant Proposal

Application ID: 16PRE27090012 R

PI Name: Zakkoyya Lewis-Powell Institution: The University of Texas Medical Branch
Project Title: TAME health: Testing Activity Monitors' Effect on health

Reviewer 1

Evaluation of the Proposal: The significance of improving cardiovascular health through increased physical activity is an important area of research that may be further informed by uses of EAMs and counseling to sustain physical activity over time. The investigator has been responsive to prior critique from reviewers. The approach is clear in the delineation of procedures. The proposal is innovative from the perspective that it encourages increased physical activity through self-monitoring + behavioral counseling using the 5A's approach. Rationale for the selection of the advanced EAM (UP25 by Jawbone) was reported. Some lingering limitations and concerns include the potential difficulties subjects may have using the UP26 EAM device app for self-monitoring.

Reviewer 2

Evaluation of the proposal

<u>Significance –</u> If the aims are achieved, the investigators can apply findings on a more widespread level that could impact clinical practice within primary care settings.

The proposal is well-designed and the reviewers' comment have been addressed. Preliminary work indicates support for the proposed study.

<u>Approach -</u>

Strengths:

Activity is important for cardiovascular health and use of electronic activity monitors pose a possible motivational method to increase physical activity in adults.

The proposal has the capacity to have relevance to a large number of US adults.

Analyses are straight-forward and ethical aspects of the proposed research are well-addressed.

Using motivational methods provides promise.

Weaknesses:

<u>Research Design –</u>

Participants – what is the rationale for the age group of 55-74? Many adults 55-74 have medical conditions that may require exclusion for unlimited activity such as coronary artery disease. The proposal does not describe including criteria that involve medical record review for co-morbid conditions.

Counseling – Who will provide the motivational counseling? “This counseling will be conducted by an experienced PA counselor with a background in exercise physiology and training in motivational interviewing.”

EAM Group – Although the proposal states that participants will be de-identified, participants may or may not want their information shared and consent and privacy issues require further discussion.

Analysis – The sample size calculation is not present. There are no calculations that provide information about whether the sample will provide effect sizes to direct the next level of study.

Innovation: The project appears original and innovative with using motivational counseling and activity monitors that may also motivate the participant.

Reviewer 3

Provided no comments on the evaluation of the proposal.
